# Use of "LIMON Test" in Constrictive Pericarditis: a case series

**DOI:** 10.1186/1749-8090-10-S1-A37

**Published:** 2015-12-16

**Authors:** Caterina Simon, Lorenzo Grazioli, Diego Cugola, Viviana Macchitelli, Francesco Innocente, Amedeo Terzi, Maurizio Merlo, Antonio M Brucato, Luca M Lorini, Lorenzo Galletti

**Affiliations:** 1 Cardiac Surgery Division, HPG 23, Bergamo, Italy; 2 Anesthesiology Department, HPG 23, Bergamo, Italy; 3 Internal Medicine HPG 23, Bergamo, Italy

## Background/Introduction

Constrictive pericarditis causes progressively impaired diastolic filling of the heart with associated symptoms of heart failure. High filling pressure in the right atrium creates high hepatic venous pressure with subsequent impairment liver function. Hepatic function has been proposed as an indirect index of cardiac failure in constrictive pericarditis. A non-invasive liver function monitoring system, "The LIMON test", has been developed to measure indocyanine green elimination (ICG) by pulse spectrophotometry. Indocyanine green hepatic clearance rate (PDR) and ICG 15-min retention (R15) are proportional to liver function.

## Aims/Objectives

The aim of this study is to evaluate the efficacy of ICG to assess the hepatic function of patients with constrictive pericarditis who are undergoing to surgical pericardiectomy.

## Method

From 2010 and 2014 ten patients with constrictive pericarditis, who underwent surgical pericardiectomy in our Institution, were enrolled in this prospective observational study. Mean age was 67 7.2 years. EF% was 57 5. Liver function was examined by "Limon Test" preoperatively and six months after surgery. Serum liver function tests were also measured on the same day.

## Results

Both right and left atrial pressures decreased after surgery (Right pressure from 17.1 1.4 mmHg to 8.7 1.5 mmHg; Wedge from 17.8 3.6 mmHg to 10.7 3.5 mmHg). PDR increased from 9.6 1.19 to 18.3 4.2 (p = 0,0156). Conversely, R15 decreased from 26.4 6.3 to 8.94 6.7 (p = 0,0016). Perioperative mortality occurred in the patient with the most low pre-operatively PDR level (2.8) and the most high level of R15 (R15=68). Serum liver function test were similar between pre and post-op: aspartate aminotransferase 34.5 12U/L vs 33 8U/L; Bilirubine 1 0.4 mg/dL vs 1.4 0.2 mg/dL; alkaline phosphatase 99 59U/L vs 77 23U/L (p = n.s.).

## Discussion/Conclusion

LIMON test can be proposed to preoperatively assess the hepatic function, instead of current liver function tests and as predictor of perioperative mortality in cardiac surgery. Larger sample size are needed in order to confirm these findings.

